# Current News

**Published:** 1892-01

**Authors:** 


### Current News.

### ANNIVERSARY MEETING OF THE FIRST DISTRICT DENTAL SOCIETY OF NEW YORK.

THE following is the programme of the Anniversary Meeting of the First District Dental Society of New York, to be held January 18, 19, 20, 1892, at the Academy of Medicine, New York City.

The essays to be read are—

"Pus Formation—Revived," by Professor D. R. Stubblefield, Nashville.

"A Plea for Replantation as a Cure for Chronic Alveolar Abscess," by Professor John S. Marshall, Chicago.

"Some Thoughts on Transformism," by Professor C. N. Peirce, Philadelphia.

"Borders between the Natural and the Artificial in Bridge- and Crown-Work," by Dr. Calvin S. Case, Jackson, Michigan.

"Adenoid Growths, Mouth-Breathing, and Thumb-Sucking, and their Relation to Irregularities of the Teeth," by Dr. Norman W. Kingsley.

Dr. Kingsley's paper will be illustrated by a number of casts of faces, models of jaws, and regulating appliances.

The following distinguished gentlemen have agreed to discuss the above papers: Dr. J. E. Garretson, Dr. C. N. Peirce, Dr. James Truman, Dr. S. H. Guilford, and Dr. Charles J. Essig, of Philadelphia; Dr. J. Taft, Cincinnati; Dr. John Marshall, Chicago; Dr. D. R. Stubblefield and Dr. J. Y. Crawford, Nashville; Dr. C. S. Stockton and Dr. S. C. G. Watkins, New Jersey; Dr. Thomas Fillebrown, Boston; Dr. E. C. Kirk, Philadelphia; Dr. G. L. Curtis, Syracuse; Professor Charles Mayr, Springfield; Dr. Frank Bliven, Worcester; Dr. F. T. Van Woert, Brooklyn; and Drs. J. N. Farrar, George Allan, and Charles Heitzmann, New York.

The following gentlemen will give new and interesting clinics: Dr. J. Y. Crawford, Nashville; Dr. George V. Brown, Duluth; Dr. Sydney S. Stowell, Pittsfield; Dr. F. T. Van Woert, Brooklyn; Dr. A. H. Gilson, Boston; Dr. John L. Gish, Jackson, Mich.; Dr. S. C. G. Watkins, New Jersey; Dr. W. F. Rehfuss, Philadelphia; Dr. S. E. Davenport, New York, and a number of others with whom we are still in correspondence. In addition, there will be a number of new instruments, devices, etc., shown by various manufacturers. The clinics will be held in the large clinic-room of the Academy, where there will be space enough for everything to be seen by all who attend.

The Trunk Line Association have agreed to sell tickets from points on their roads for one fare and a third on the following conditions:

(1) That at least one hundred persons who have travelled to the meeting on some legitimate form of railroad shall be in attendance; which fact shall be certified to by the secretary of the society, and also by a special agent of the railroads who will be in attendance.

(2) That all other provisions written on the back of the certificates shall be complied with.

Persons desiring to attend may bring with them any member of their immediate family. They must pay full fare to New York, and be sure to take a receipt in the form of a certificate from the ticket agent where they purchase ticket. This certificate, when signed, will entitle holder to return for one-third fare.

The profession are cordially invited to attend.

RODRIGUES OTTOLENGUI,
*Chairman Executive Committee.*

115 MADISON AVENUE, N. Y.

### FIRST DISTRICT DENTAL SOCIETY, NEW YORK—SPECIAL ANNOUNCEMENT.

A MASS-MEETING of all interested in the Dental Protective Association will be held Wednesday morning during the anniversary. Dr. Crouse, of Chicago, will preside.

R. OTTOLENGUI.

---

### AMERICAN ACADEMY OF DENTAL SCIENCE.

THE Twenty-fourth Annual Meeting and banquet of the American Academy of Dental Science was held at the Copley Square Hotel, Boston, November 11, 1891, about twenty-five members being in attendance. The meeting was called to order at 4 P.M. by the President, Dr. F. N. Seabury, of Providence, R. I. The annual reports were read and accepted, and the following-named officers were elected for the ensuing year.

President, Dr. Charles A. Brackett, of Newport, R. I.; Vice-President, Dr. Eugene H. Smith, of Boston; Recording Secretary, Dr. Charles H. Taft, of Cambridge; Corresponding Secretary, Dr. Edward N. Harris, of Boston; Treasurer, Dr. Virgil C. Pond, of Boston; Librarian, Dr. Edward C. Briggs, of Boston; Editor, Dr. William H. Potter, of Boston.

*Executive Committee.*—Dr. F. E. Banfield, of Boston; Dr. F. N. Seabury, of Providence; Dr. Thomas Fillebrown, of Boston.

After a vote of thanks had been extended to the retiring President, an interesting paper, entitled "Methods of filling Teeth Forty Years Ago," was presented by Dr. Seabury, followed by a paper by Dr. George C. Ainsworth describing a method of regulating upon the spring-band principle.

Following the discussion of the papers, the Academy gave its attention to the orator of the evening, Dr. George S. Allan, of New York, whose address was listened to with marked attention, and at the close of which a vote of thanks was passed and a copy requested for publication. At the request of several members of the Academy, Dr. Allan then related in a brief and informal manner some of his experiences in the use of bichloride of mercury as a germicide, upon the conclusion of which adjournment was made for the annual dinner.

The newly-elected president, Dr. Brackett, in a few well-chosen words, opened the post-prandial exercises, which continued until eleven o'clock, when final adjournment was made.

Charles H. Taft,
*Recording Secretary.*

15 Brattle Street, Cambridge.

---

The California State Board of Dental Examiners has had presented to it for endorsement a diploma bearing the heading of the "Medical University of Ohio." The diploma reads as follows:

"The Medical University of Ohio.
"Special
"Diploma.

"This certifies that John D. Van Vleck, M D., having successfully passed a special examination in the Department of Dentistry and Oral Surgery, is qualified to practise in the above departments.

"In testimony whereof we, the Professors in said departments and the President of the University, do hereby affix our names and the seal of the University of Cincinnati, Ohio, this 1st day in the month of March, in the year of our Lord MDCCCLXXXVIII.

"B. F. EVANS, D.D.S., M.D.
"CHARLES BRADLEY, M.D.,
Professor of Anatomy.
"G. W. VAN VLECK, A.M., M.D.,
President of the University.
"JOHN M. DIXON, M.D., Secretary."

Is any one familiar with such a university or department of dentistry?

---

Election of Officers of the New England Dental Society.—The following were elected officers of the New England Dental Society, at its last meeting, held October 29 and 30, 1891, for the ensuing year:

President, Dr. J. F. Adams, Worcester, Mass.; Vice-Presidents, Dr. S. G. Stevens and Dr. J. H. Daly, Boston, Mass.; Secretary, Dr. Edgar O. Kinsman, Cambridge, Mass.; Assistant Secretary, Dr. J. H. McQuade, Medford, Mass.; Treasurer, Dr. George A. Young, Concord, N. H.; Librarian, Dr. A. H. Gilson, Boston, Mass.

*Executive Committee.*—Dr. W. E. Page, Boston, Mass.; Dr. George F. Cheney, St. Johnsbury, Vt.; Dr. C. H. Hayward, Peterborough, N. H.; Dr. W. P. Cooke, Boston, Mass.; Dr. Marion L. Woodward, Boston, Mass.

Edgar O. Kinsman,
*Secretary.*

